# Exploration of Voids, Acoustic Properties and Vibration Damping Ratio of Cyperus Pangorei Rottb Fiber and Ramie Fiber Reinforced with Epoxy Resin Hybrid Composites

**DOI:** 10.3390/polym16060832

**Published:** 2024-03-18

**Authors:** Sudhakar Kanniyappan, Senthil Kumaran Selvaraj

**Affiliations:** School of Mechanical Engineering, Vellore Institute of Technology, Vellore 632014, India; sudhakar2info@gmail.com

**Keywords:** Cyperus pangorei rottb, ramie, vacuum resin infusion process, computed tomography scan, impedance tube, natural frequency, damping

## Abstract

Noise pollution is a major threat to the health and well-being of the entire world; this issue forces researchers to find new sound absorption and insulating material. In this paper, the sound absorption coefficient and vibration damping factor of panels manufactured from Cyperus pangorei rottb and ramie fiber reinforced with epoxy resin are explored. Cyperus pangorei rottb grass fiber and ramie fiber are widely available natural fibers. Cyperus pangorei rottb grass fiber is used in mat manufacturing, whereas ramie is widely used as a fabric. Using both of these fibers, six variant panels using a vacuum resin infusion process (VRIP) were fabricated. The panels were named C, R, CR, RCR-Flat, RCR-Curved, and RCR-Perforated. All the panels were tested for the sound absorption coefficient using an impedance tube with a frequency ranging up to 6300 Hz. Modal analysis was carried out by using the impulse hammer excitation method. A micro X-ray computed tomography (CT) scan was used to study the voids present in the panels. The results were compared among the six variants. The results show that the RCR-curved panel had the highest sound-absorbing coefficient of 0.976 at a frequency range between 4500 Hz to 5000 Hz. These panels also showed better natural frequency and damping factors. The presence of internal voids in these panels enhances sound absorption properties. These panels can be used at higher frequencies.

## 1. Introduction

Sound or noise pollution, which refers to the unwanted and excessive sounds that surround us, is not just an irritant. It is a growing threat to our physical and mental well-being. From the rumble of traffic to blaring music, constant exposure to noise can trigger a cascade of negative effects such as hearing loss, cardiovascular disease, sleep disturbance, stress, and anxiety. In order to overcome these noise problems, sound-absorbing and -insulating panels or materials are used. Generally, these materials are fabricated from synthetic fibers such as polymer foams, wool, glass, fabric filler, and polymer fibers, posing additional harm to the environment and being more expensive. Instead of relying on synthetic materials for soundproofing/absorbing, we can turn to the wonders of nature [1]. Enter the world of natural fibers like ramie, flax kenaf, sisal jute, cotton, flax, sisal, and hemp—all superstars in the realm of sustainability. Grown from renewable resources, these wonder-fibers offer a triple threat: affordability, biodegradability, and recyclability.

Let us take a quick look at ramie fiber and Cyperus pangorei rottb. The modest “Mat Sedge”, Cyperus pangorei rottb, rises from the lush embrace of marshes and graces the banks of rivers. This fiber is a lignocellulosic fiber that contains lignin (17.88%) and cellulose (68.5%), with a small quantity of other components [2,3,4]. At first glance, it might seem like any other ordinary plant. However, beyond its modest façade is a wealth of untapped potential just waiting to be discovered. This hardy sedge has been woven for ages into the fabric of human cultures and was originally from the subtropical regions of Asia. Its uses go far beyond its ecological function in wetland ecosystems, exhibiting a startling diversity that includes fine workmanship, conventional medicine, and even environmentally friendly solutions for the contemporary world. Weaving mats using this material is a common practice.

The natural bast fiber called ramie fiber is taken from the stems of the Boehmeria nivea plant. It contains cellulose (68.5–85%), hemicellulose (13.16.7%), and lignin (0.5–0.7%) [5]. It is renowned for having remarkable luster, strength, durability, low weight, and inherent flame resistance [6,7,8,9]. Ramie fibers are superior sound insulators and absorbers. They work well to stop noise from passing through ceilings, floors, and walls because of their capacity to absorb and attenuate sound waves. They are perfect for use in noise reduction applications because they can efficiently absorb sound waves across a wide range of frequencies.

Dakai Chen et al. [10] studied the acoustic properties of ramie fiber with special structures, concluding that their sound absorption properties were enhanced. Hyunjin Cho et al. [11] concluded that ramie fiber is a multipurpose material. Gokul Kumar et al. [12] found that panels made of ramie fiber with other natural waste fillers boost sound absorption properties enormously. Ramie fibers are also used as an insulating panel [13]. Kalaiselvi et al. [14] evaluated the sound absorption properties of Cyperus pangorei rottb by fully insulating a room with a Cyperus pangorei rottb fiber mat (pattamadai mat), ultimately giving credit to the material. Benazir et al. [15] studied the properties of Cyperus pangorei rottb. The addition of ramie fibers in a composite gives a good vibration damping effect [16]. At present, the void content of the composites has been successfully studied using a CT scan, which is a non-destructive method [17,18,19,20]. A CT scan was used to study cavity or void geometry, which helped to enhance the sound absorption property of rice and bulk wheat husks [21] Xiaodong Xu et al. [22] investigated the fracture process of polymer composites by using a CT scan.

The objective of this study is to develop and characterize the properties of sound-absorbing panels made from Cyperus pangorei rottb fiber/ramie fiber reinforced with epoxy composites. These panels can be used as a sound absorber as well as interior decorating panels in auditoriums, concert halls, theatres, and industries. Six different panels were fabricated by combining Cyperus pangorei rottb fiber/ramie fiber/epoxy resin using the vacuum resin infusion process. The acoustic and vibration damping of composites were investigated. Additionally, the void content of the composites was investigated using a powerful tool, a micro X-ray CT scan, for the composite laminates.

## 2. Materials and Methods

### 2.1. Materials

The materials used for the fabrication of the panels were Cyperus pangorei rottb fiber, ramie fiber, and epoxy resin. Cyperus pangorei rottb fiber was purchased from M/S Sri Balaji Mat Industries, Agaramcheri (Village & post), Vellore, Tamil Nadu, India-635804. A unidirectional ramie fiber in mat format was purchased from M/S Go Green Products, Chennai, Tamil Nadu, India-600087. Epoxy resin (LY 556) and hardener (HY 951) were purchased from M/S Covai Seenu & Company; Coimbatore, Tamil Nadu, India-641012.

### 2.2. Fabrication of Panels

The vacuum resin infusion process (VRIP) is used to fabricate the panels. Vacuum resin infusion is a composite fabrication technique renowned for its remarkable efficiency and near-flawless results [23]. Unlike traditional hand layup methods, VRIP eliminates the mess and air bubbles associated with brushing resin onto reinforcements. This method is one of the best methods to prepare composite laminates with less defects. The ramie fibers, which are in mat format, are arranged one over the another on a VRIP mold board. Before arranging the fibers, wax is applied on the board as a coating in an amount as much as is required for easy removal of the cured laminate. After arranging the fibers, it is covered by peelply, which helps to remove the laminate easily after curing. The peelply separates the infusion mesh and the fiber layers. The infusion mesh helps the resin to spread in a uniform manner. Finally, using a vacuum bagging sheet, the fibers, peelply, and infusion mesh are sealed with sealant tape on all four sides of the setup board. Spiral tubes are placed opposite to each other or as required. One end of one of the spiral tubes is connected to an inlet pipe of resin, whereas another spiral tube is connected to the inlet of the vacuum pump inlet tube.

**Figure 1:**
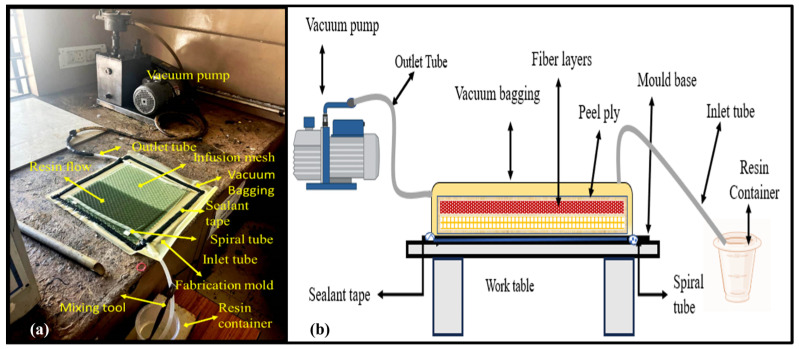
(**a**) Experimental setup; (**b**) schematic diagram of the vacuum resin infusion process.

The entire setup is sealed properly using sealant tape so that no air passes into the VRIP setup. Then, the vacuum pump is switched on by closing the resin inlet tube using a stop valve. The pressure of the 0.6 bar is maintained throughout the process. Negative pressure is created inside the vacuum bagging sheet; then, the resin inlet valve is opened to allow the resin to flow. When the resin fills or is spread over the entire area of the fiber, the inlet valve is closed inside the setup. The vacuum pump is also turned off by closing the outlet valve. Both the inlet and outlet tube of the setup has to be closed firmly or sealed completely. The setup is left for curing. The curing time is 24 h at room temperature. The size of the fabricated laminate is 280 × 280 × 5 mm. From this, laminate specimens are cut to required dimensions. The experimental setup and schematic representation of VRIP is shown in Figure 1.

**Figure 2:**
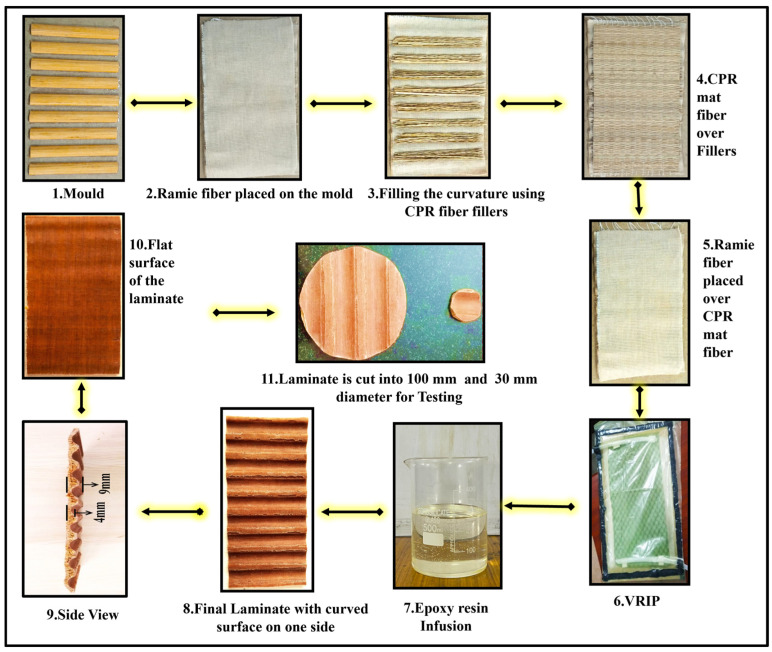
Fabrication of curved surface panel.

The sample (C) is fabricated by using two layers of Cyperus pangorei rottb grass fiber in a mat format by reinforcing it with epoxy resin. The second sample (R) is fabricated by using ramie fiber. The third sample (CR) is the combination of both Cyperus pangorei rottb and ramie fiber. The fourth sample (RCR-Flat) is fabricated by placing the Cyperus pangorei in between the two layers of ramie fiber on either side. The fifth sample (RCR-Flat) is fabricated in a manner to have a flat surface on one side and a curved surface on the other side, as shown in Figure 2. To fabricate this sample, a separate pattern is prepared using a quarter-circle carpentry wooden beading stick with a base measuring 18 mm and a curvature height of 5 mm. The wooden beading stick is cut into required sizes, and a pattern is created by placing each stick parallel to each other with a distance of 10 mm over a flat surface cardboard. Then, the pattern is used for fabrication similarly to previous methods. The sixth sample (RCR-Perforated) is prepared by drilling holes measuring 2.5 mm in diameter and 4 mm in depth over the curvature peak surfaces randomly in the fifth sample itself, as shown in Figure 2. Table 1 shows the dimensions of specimens used for sound absorption coefficient measurements.

### 2.3. Experimental Studies

#### 2.3.1. Sound Absorption Coefficient (SAC)

**Figure 3:**
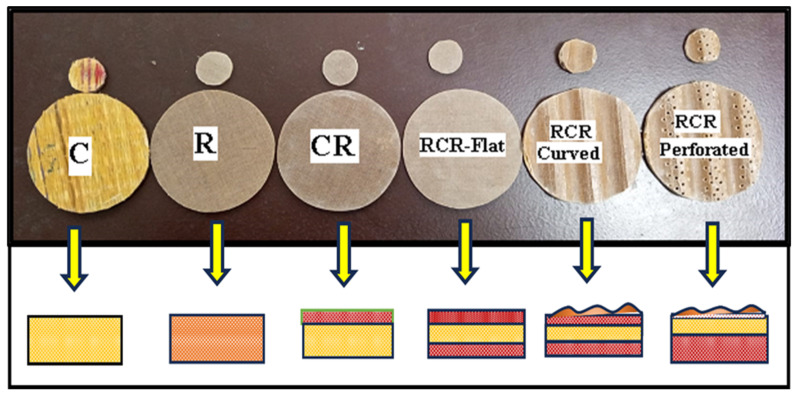
Schematic view of the samples/specimens used for testing in an impedance tube.

**Figure 4:**
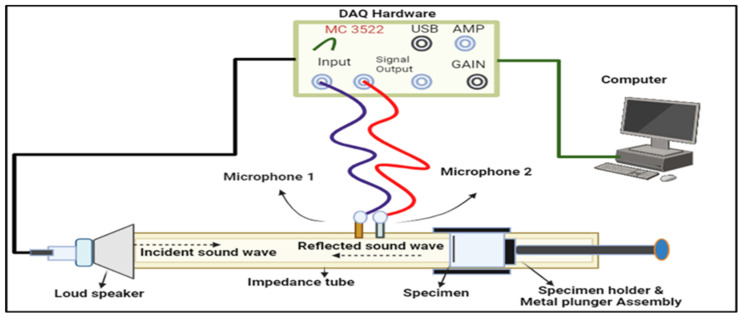
Schematic diagram of the impedance tube.

**Figure 5:**
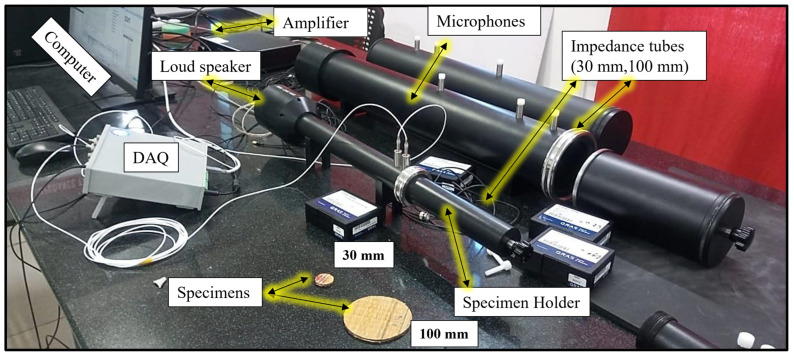
Impedance tube experimental setup.

Figure 3 shows a schematic view of the fabricated panels with the dimensions in Table 2. These fabricated panels are tested as per the ASTM E1050-08 standard [24] using an impedance tube. The transfer function method, which uses two microphones to capture sound produced by a nearby source, is used to analyze the incident and reflected waves to calculate the sound absorption coefficient. To ensure accuracy, two different tube diameters (100 mm and 30 mm) are employed for different frequency ranges (63–2000 Hz and 500–6300 Hz, respectively), along with temperature and humidity control (25 °C and 60%). Additionally, to minimize microphone phase discrepancies, the positions of the microphones are swapped during each measurement. The impedance tube schematic diagram and its setup are shown in Figure 4 and Figure 5.

#### 2.3.2. Modal Analysis (Free Vibration Test)

**Figure 6:**
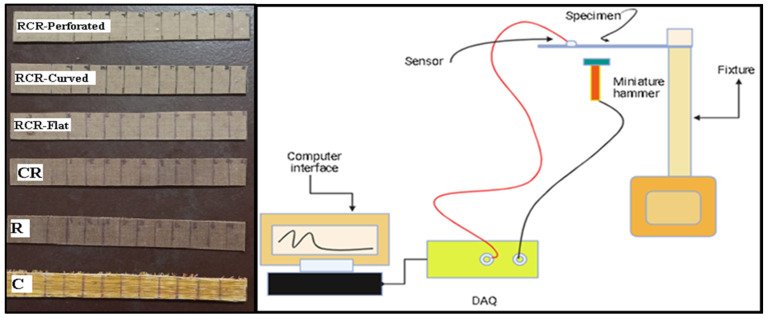
Test specimens and schematic diagram of the modal analysis test setup.

**Figure 7:**
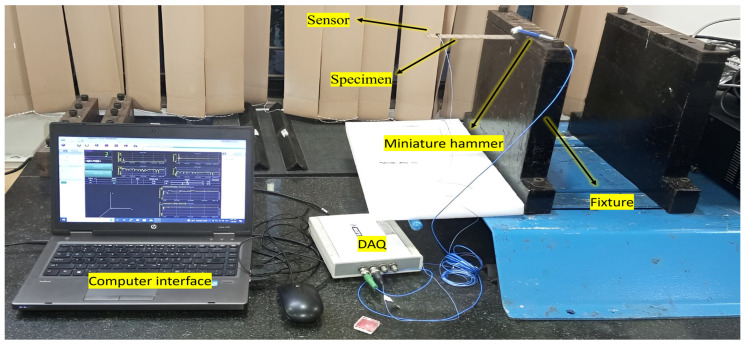
Experimental setup of the modal analysis.

An impulse hammer excitation [16] test is performed on the fabricated samples in accordance with the recommendations outlined in ASTM E756 [25]. A free vibration test is performed to find the natural frequency and damping factors. A free vibration test setup, along with the specimens and the schematic diagram, is shown in Figure 6 and Figure 7. Using fixtures, the specimens are arranged to resemble a cantilever beam construction. The equipment used are miniature impact hammer (Make: Dytran, Model—5800SL, S/N-8224, Ref.Sensitivity-104 Mv/Lb), miniature accelerometer Sensor (352C22/NC, S/N-LW282645), and data acquisition (DAQ) card, which is connected to a PC and interfaced with Dewesoft 7.1 (https://downloads.dewesoft.com/dewesoftx-actual/DewesoftX_2024.1_240220_x64.exe (accessed on 18 January 2024)). The frequency response function (FRF) curve obtained is analyzed, and the values of the natural frequency and damping factors are recorded.

#### 2.3.3. Void Measurements Using CT Scan

Usually, void volume fraction—also known as void content—is used to describe voids in laminates [26]. The gravimetric method (density measurement), ultrasound attenuation, and microscopy are familiar methods to measure voids. Out of these, only the microscopy method provides images that are required for void characterization. However, it is only capable of scanning “2D slices” of the material, and it is a destructive, lengthy process. Therefore, in this study, micro X-ray CT scanning was utilized as the primary tool for void characterization, as it is a non-destructive testing method. The best acoustical property sample was scanned in order to study its internal structure. A Skyscan1273/Bruker CT scanner was used to scan the samples. A source voltage of 80 kv and a source current of 187 μA were used with three images per projection. The void content was analyzed using CTAN software (Bruker CTAn v.1.18 Micro-CT Software|Blue Scientific (blue-scientific.com (accessed on 15 February 2024))), and images were analyzed with CT Vox software (Version: 1.20.8.0) (CTVox Micro-CT Volume Rendering Software|Blue Scientific (blue-scientific.com (accessed on 15 February 2024))).

## 3. Results and Discussion

### 3.1. Sound Absorption Coefficient (SAC)

**Figure 8:**
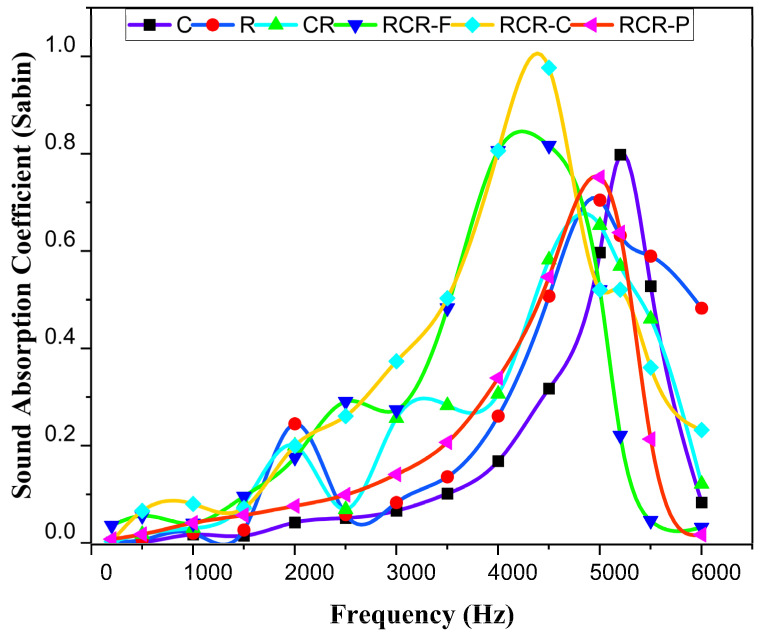
Sound absorption coefficient of C, R, CR, RCR-Flat, RCR-Curved, and RCR-Perforated panels.

Figure 8 shows the SAC of all the panels. It is to be noted that the SAC of the RCR-Curved panel is superior to that of other panels, with frequencies ranging between 4000 Hz to 5000 Hz. The sound absorption coefficient for the CR panel also sounds good in comparison with other panels.

**Figure 9:**
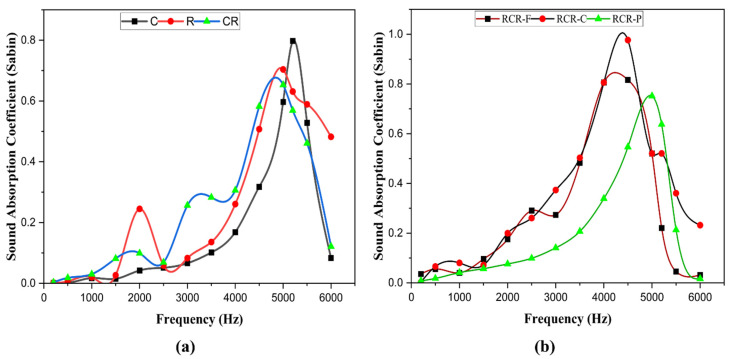
SAC split up: (**a**) C, R, and CR panels; (**b**) RCR-Flat, RCR-Curved, and RCR-Perforated panels.

The SAC of C is greater than that of the R and CR panels in frequencies between 4500 Hz to 5500 Hz. The C panel has greater SAC than R in the region of frequencies ranging between 1000 Hz and 5000 Hz. The C, R, and CR panels have the highest SAC of 0.798, 0.704, and 0.653, respectively, from 5000 to 5200 Hz which is shown in Figure 9a. Similarly, the SAC of RCR-Flat and RCR-Curved panels have values of 0.816 and 0.976 with a frequency range of 4000 Hz to 5000 Hz, whereas RCR-Perforated panels have a 0.751 value at 5200 Hz, respectively as shown in Figure 9b. The results show that these panels can be used as sound-absorbing materials at higher frequencies. The reason behind this is that ramie fiber is made up of around 70% of holocellulose; it is said that materials that have 60 to 70% of the holocellulose component are good for sound absorption [27]. As both the materials utilized here have a good percentage of holocellulose, they absorb sound very well. The Cyperus pangorei rottb fiber is placed between the two layers of ramie fiber as a filler, as well as in mat format.

**Figure 10:**
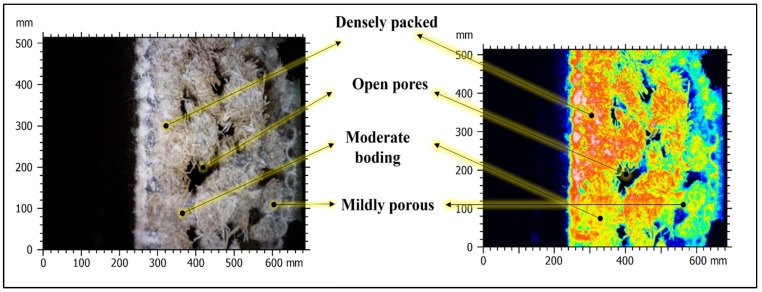
Digital microscope image shows the internal structure of the RCR-Curved panel.

When the cut structure is analyzed under a digital microscope, it is possible to see open voids between the fibers, which is shown in Figure 10. The red color indicates that the fibers are densely packed and bonded very well. The black color shows the open pores. The yellow color represents moderate bonding and mild porosity. The light green color and blue color represent highly porous structures. When the CT scan is taken, it is possible to see that are many closed cavity/pores found inside the panel, which are called voids. The presence of these voids allows the panels to absorb sound waves. The sound waves reverberate internally inside the voids, and energy in the form of kinetic energy is converted into heat, which is lost to the atmosphere [28,29].

### 3.2. Analysis of Natural Frequency

**Figure 11:**
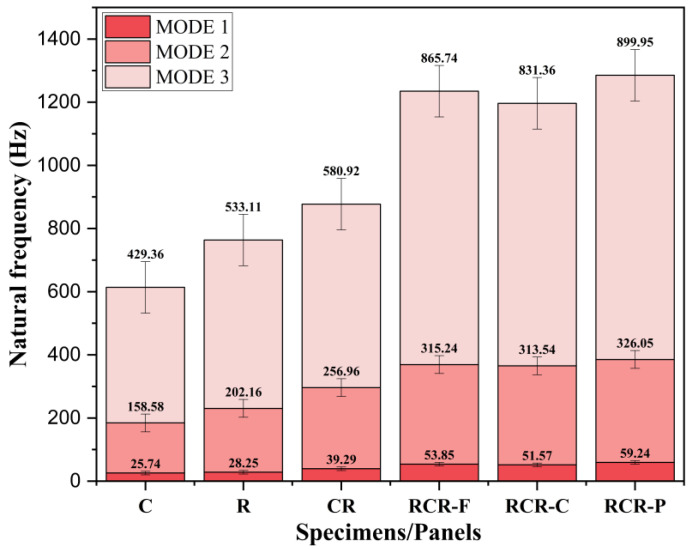
Modal analysis histogram.

The specimen to be fixed in the fixture is to be in the form of a cantilever. The specimen is impacted at 11 equally spaced points by a miniature impact hammer. In the time domain, the three parameters, which include displacement signal, force, and magnitude, are measured. Then, the data are processed using Dewesoft 7.1 to obtain the frequency response function (FRF) curve for all the composite panels with different hammer points. The three modes are bending (Mode 1), twisting (Mode 2), and bending (Mode 3). Natural frequency is recorded using the peak point in each curve that is evident from the frequency response function (FRF). In Figure 11, the natural frequencies of different specimens are shown. It can be seen that the natural frequency increases rapidly for each panel.

In mode 1, panel R shows an increase of 9.75% in comparison to the C panel, the CR panel shows a 39% increase in comparison to the R panel, the RCR-Flat panel shows an increase of 37.05% in comparison to the CR panel, the RCR-Curved panel shows a decrease of 4.23% in comparison to the RCR-Flat panel, and the RCR-Perforated panel shows an increase of 14.87% in comparison to the RCR-Perforated panel. Similarly, in mode 2 and mode 3, the same strategy follows. Natural frequency increases with the reduction in mass. Higher stiffness also leads to an increase in natural frequency. It can be observed that the higher frequency panel has many voids in comparison to other panels.

### 3.3. Analysis of Damping Factor

**Figure 12:**
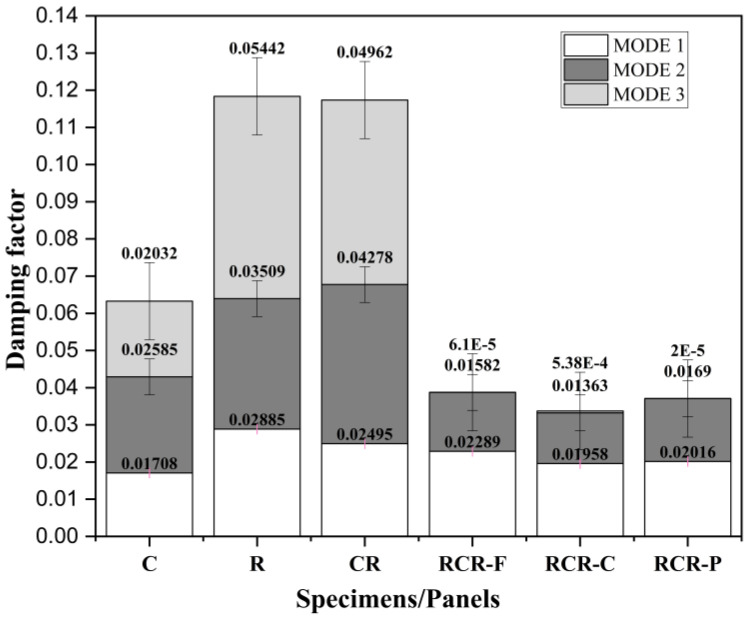
Damping factors of the panels in three modes.

The damping ratios of the different panels were recorded from the frequency response function (FRF) curve, which is shown in Figure 12. The ramie fiber, compared with the Cyperus pangorei rottb grass fiber, has better elasticity, stiffness, strength, and durability properties, which are added advantages to the panel. The ramie fiber panel in mode 3 has the highest damping factor, whereas the other hybrid panels such as C, CR, RCR-Flat, RCR-Curved, and RCR-Perforated show values less than that of the ramie fiber. But the average damping factor for all of the panels at all of the modes indicates that the CR panel has a good damping factor. In mode 1, panel R has highest damping factor due to higher stiffness and fibers’ strong interfacial bonding with the matrix. In mode 2, the CR panel exhibits a higher damping factor, which is the combined effect of both of the fibers. The fiber molecules are densely packed when the epoxy resin combines both of the fibers externally, but internally, there is a presence of voids, which supports damping in Cyperus pangorei rottb fiber indirectly. The wax [30] content present in the fiber segregates the resin from the fiber, which affects the properties. Similarly, in mode 3, panel R again has the highest damping factor. A rise in natural frequency results in a reduction in the damping ratio. Similar behavior was observed by Kumar et al. [31] for banana/polyester and sisal/polyester composites, as well as by Etaati et al. [32] for hemp/PP composites. Ramie fibers, when used as a hybrid composite, enhance vibrational properties [33,34,35].

### 3.4. Voids Analysis 

**Figure 13:**
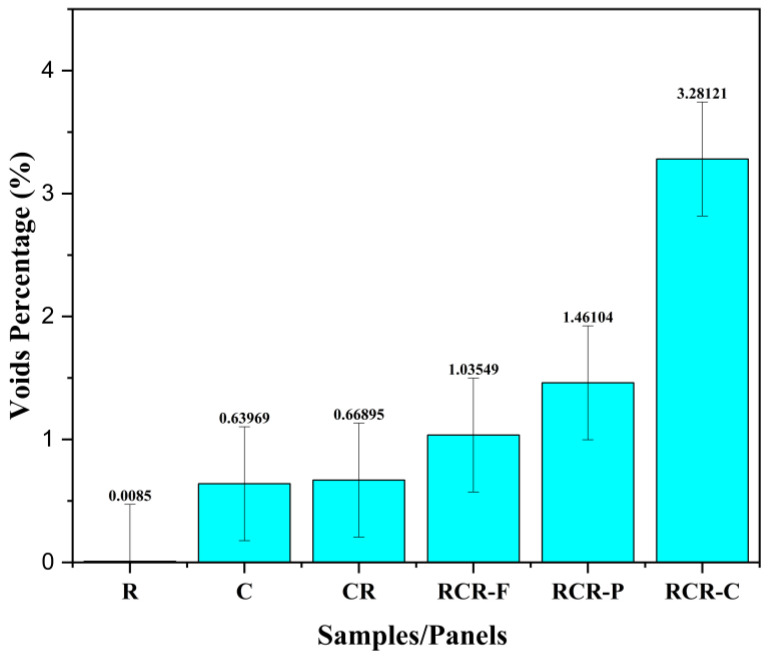
Void content of the panels, measured in percentages.

The CT-scanned images are solely used to estimate the void content of the material [36,37,38]. It is generally acknowledged that there is a correlation between areal and volume fractions for high-volume fractions of uniformly dispersed voids and/or exceptionally large scanned regions. The presence of internal voids is presented in percentages, which is shown in Figure 13 below. The best acoustical performance panel has the highest percentage of internal voids (RCR-C-3.28121%). The presence of voids is due to epoxy resin flow under vacuum conditions. In VRIP, the fibers become compressed one over the other due to negative pressure; in the meantime, when the resin is allowed to flow, it spreads as fast as it can, which leads to voids in between the layers of the fibers. These voids are an added benefit for SAC. Generally, the sound waves that enter into the closed cavity reverberate inside the cavity itself and lose their energy in the form of heat to the surrounding environment.

**Figure 14:**
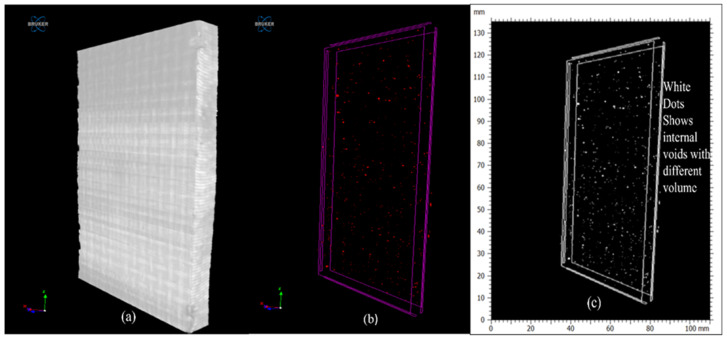
CT-scanned image of a ramie fiber panel with internal voids.

In Figure 14, we can see that there is a presence of internal voids with different volumes in the panel. In Figure 14a, which shows a CT-scanned image, neither porosity nor voids are visible in the top surface. But when analyzed by slicing and by changing the view using CTAN software, as seen in Figure 14b,c, an image appears with red dots/white dots, which shows voids distributed everywhere in the panel.

**Figure 15:**
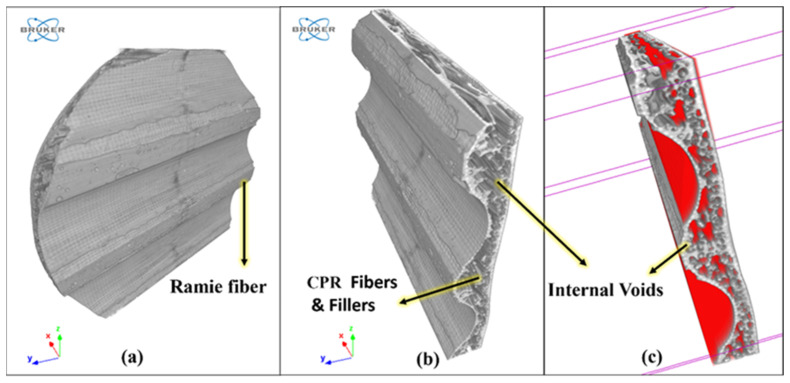
CT-scanned image of the surface of an RCR-Curved panel with internal voids.

Figure 15a shows a CT-scanned image of the surface of the RCR-Curved panel. When the internal structure is viewed by slicing the scanned image using CTAN V 1.18 software, it is possible to see a large cavity situated internally between the fillers, as is shown in Figure 15b. In Figure 15c red color shows the internal cavity or voids along with fibers. The resin did not bond the internal Cyperus pangorei rottb fillers completely. Instead, the upper layer and the bottom layers, which are ramie mat fibers, were bonded completely. This creates closed internal voids. These internal cavities/voids are seen as one of the reasons for the enhanced sound absorption or acoustic properties of this material. The voids that are present internally inside the panels absorb the longitudinal sound waves and convert them to heat where they lose their energy. As a result, this results in the absorption of sound. As the heat energy is minimal, and because the panels are also in contact with the air, the heat is balanced, with practically no effect on the panel.

**Figure 16:**
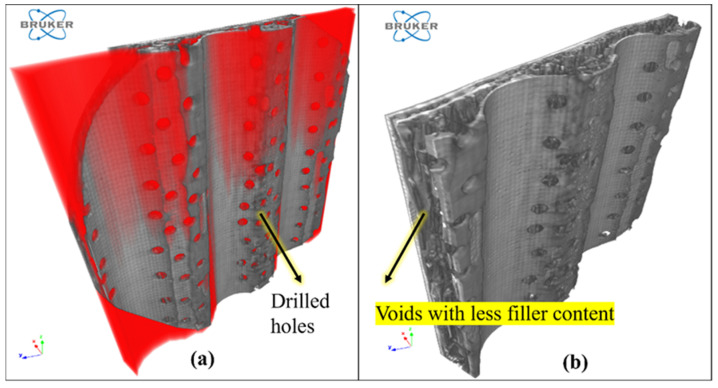
CT-scanned image of RCR-Perforated panel.

Figure 16 shows the scanned image of an RCR-Perforated Pannel. The red color in Figure 16a shows the presence of voids. When the image is sliced using CTAN V 1.18 software, it is possible to observe that the panel contains many drilled holes and internal voids in Figure 16b. These voids are open voids, and this does not enhance the sound absorption properties but enhances natural frequency. The reason for this may be that when perforated manually, using a drilling machine, the filler content inside the panel is removed.

**Figure 17:**
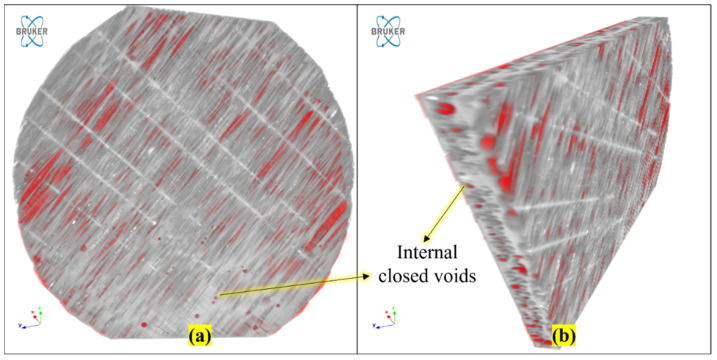
CT-scanned image of Cyperus pangorei rottb fiber panel with internal voids.

Figure 17 shows a scanned image of Cyperus pangorei rottb fiber panel. When the image is sliced in a surface direction (Figure 17a) as well as in the cross section (Figure 17b) of the panel, the internal voids are seen with a different geometry. On the top surface, cavity spots are identified as small dots as well as in large size. The cross section shows the internal cavities (closed voids) in red color which is seen at the edges of the panel.

## 4. Conclusions

Cyperus pangorei rottb fiber and ramie fiber hybrid composite panels of six variants were successfully prepared by using a vacuum resin infusion process (VRIP) and then characterized. The structure of the RCR-Curved panel had an SAC value of 0.976, whereas the RCR-Flat panel had an SAC value of 0.816. The other panels also had good sound absorption performance. A computed tomography (CT) scan showed the presence of voids. The panels demonstrated better natural frequency and damping factors, whereas the panel R dominated. The high chemical content of holocellulose in both the fibers and the presence of internal voids enhanced the sound absorption coefficient. These panels can play a dual role, such as a sound-absorbing material at higher frequencies and a lightweight material for interior decorations. Therefore, panels fabricated from Cyperus pangorei rottb fiber and ramie fiber are suitable for applications where the damping factor and sound absorption at higher frequencies are significant design criteria.

## Figures and Tables

**Table 1:** Specimen dimensions.

Specimen Name	No. of Layers of Reinforcement	Thickness (mm)	Diameter (mm)
C	2	5	30, 100
R	8	5	30, 100
RC	C-1, R-4	5	30, 100
RCR-Flat	C-1, R-4	5	30, 100
RCR-Curved	C-1, R-4, C-Fillers	4 & 9	30, 100
RCR-Perforated (2.5 mm)	C-1, R-4, C-Fillers	4 & 9	30, 100

**Table 2:** Dimensions of free vibration test specimens.

Specimen Name	No. of Layers of Reinforcement	Length (mm)	Thickness (mm)
C	2	200	5
R	8	200	5
RC	C-1, R-4	200	5
RCR-Flat	C-1, R-4	200	5
RCR-Curved	C-1, R-4, C-Fillers	200	4 & 9
RCR-Perforated (2.5 mm)	C-1, R-4, C-Fillers	200	4 & 9

## Data Availability

Data is available on the request.
